# A Secure and Robust Image Hashing Scheme Using Gaussian Pyramids

**DOI:** 10.3390/e21111132

**Published:** 2019-11-19

**Authors:** Iram Bashir, Fawad Ahmed, Jawad Ahmad, Wadii Boulila, Nouf Alharbi

**Affiliations:** 1 Department of Electrical Engineering, HITEC University Taxila, Punjab 47080, Pakistan; engrirambashir@gmail.com (I.B.); fawad@hitecuni.edu.pk (F.A.); 2 School of Computing, Edinburgh Napier University, Edinburgh EH10 5DT, UK; J.Ahmad@napier.ac.uk; 3 College of Computer Science and Engineering, Taibah University, Al-Madinah P.O. Box 344, Saudi Arabia; nmoharbi@taibahu.edu.sa; 4 RIADI Laboratory, University of Manouba, Manouba 2010, Tunisia

**Keywords:** hash, digital image, security, distortions, False Positive Probability (FPP), False Negative Probability (FNP)

## Abstract

Image hash is an alternative to cryptographic hash functions for checking integrity of digital images. Compared to cryptographic hash functions, an image hash or a Perceptual Hash Function (PHF) is resilient to content preserving distortions and sensitive to malicious tampering. In this paper, a robust and secure image hashing technique using a Gaussian pyramid is proposed. A Gaussian pyramid decomposes an image into different resolution levels which can be utilized to obtain robust and compact hash features. These stable features have been utilized in the proposed work to construct a secure and robust image hash. The proposed scheme uses Laplacian of Gaussian (LOG) and disk filters to filter the low-resolution Gaussian decomposed image. The filtered images are then subtracted and their difference is used as a hash. To make the hash secure, a key is introduced before feature extraction, thus making the entire feature space random. The proposed hashing scheme has been evaluated through a number of experiments involving cases of non-malicious distortions and malicious tampering. Experimental results reveal that the proposed hashing scheme is robust against non-malicious distortions and is sensitive to detect minute malicious tampering. Moreover, False Positive Probability (FPP) and False Negative Probability (FNP) results demonstrate the effectiveness of the proposed scheme when compared to state-of-the-art image hashing algorithms proposed in the literature.

## 1. Introduction

In recent years, there has been tremendous advancement in multimedia technologies that encompasses images, video and audio [1,2,3]. As a result, it has become easy to create, broadcast, distribute and store digital content. A number of tools are easily available through which tampering of digital media can be done very effectively [4,5]. This poses a serious problem in case a digital content is to be used as an evidence. To address this issue, digital watermarking and hashing techniques have been proposed. A digital watermark is an imperceptible signal added to a digital content for integrity verification and copyright protection. The disadvantage of using a watermark is the extra payload added to the original content. Hashing is an alternate way to check the integrity of digital content. Cryptographic hash functions such as SHA1 generates a fixed size 160-bit code which can be used for integrity verification and authentication. An important property of cryptographic hash is its sensitivity to the change in input data. For example, hash generated through SHA1 completely changes even if a single bit of input data is changed.

Despite successful use of cryptographic hash functions in e-commerce and other applications, researchers have developed novel hashing algorithms for multimedia content verification during the past two decades. This is because of the difference between text data and multimedia data. For text data, a single bit change in data would change the entire meaning whereas in case of multimedia data, a single bit change in data value generally keeps the semantic of the content intact. Therefore, for integrity verification of multimedia data like digital images, a robust hash function is required. Image hashing is a process which converts the actual input image into a short numeric representation. Image hash is also referred to as a Perceptual Hash Function (PHF). To obtain hash of an image, spatial or transform domain features are extracted from the image and used to generate its hash [6]. The selection of feature is an important aspect in image hashing. Features should be chosen such that they are robust to content preserving or non-malicious operations such as compression, filtering, brightness contrast adjustment, geometric transformation, etc., and should change when the image undergoes malicious tampering. In addition, the hash should be made secure against hash collision and other attacks by using one or more secret keys. The hash should also be compact in size.

**Figure 1:**
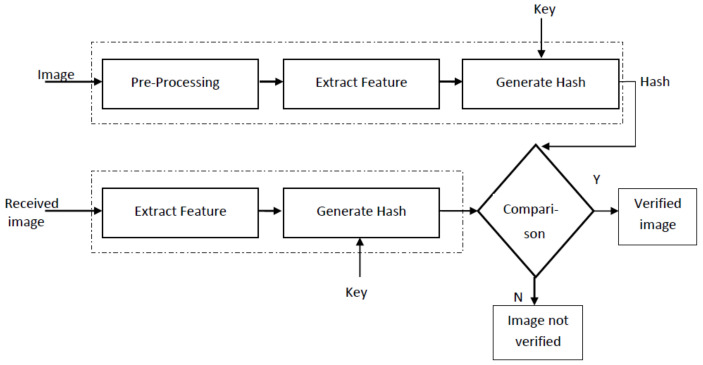
General block diagram of an image hashing scheme.

The general framework of image hashing is shown in Figure 1. The input image is first pre-processed before feature extraction. Pre-processing generally involves image resizing, color to gray-scale conversion and low-pass filtering to remove unwanted noise. Relevant features are then extracted from the pre-processed image. The type of feature selected has a very significant impact on robustness and tamper detection capability of an image hashing scheme. The extracted features are processed to form image hash. This step may additionally involve compression of features so that the size of the hash is considerably reduced. For image integrity verification, the generated hash and secret key is usually transmitted to the receiver through a secure channel, while the image be may transmitted using an insecure channel. The hash of the received image is calculated and compared with the received hash. If both the hashes match, the received image is declared authentic. Different comparison metrics can be used; for example, Hamming distance, bit-error rate, Euclidean distance, sum of absolute difference, etc. To make the hash secure against intentional attacks, a secret key is used either in the hash generation or the feature extraction stage. The purpose of using secret key is to make it extremely difficult for an attacker to produce correct hash for a given input image.

Following are some important properties of a PHF [7]:

- A PHF should be robust to non-malicious distortions like compression, Gaussian noise, motion blurring, etc.
- A PHF needs to be sensitive to tampering along with tamper localization.
- A PHF should be key dependent. Without knowledge of the secret key, it should be extremely difficult to calculate the correct hash.

**Figure 2:**
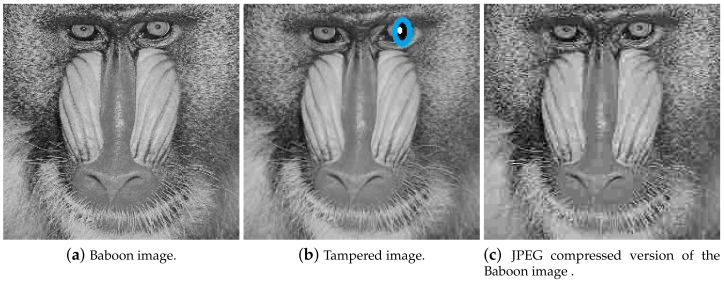
Illustration of malicious and non-malicious distortions.

To explain the first two points, consider the Baboon image shown in Figure 2a and its tampered version in Figure 2b. Tampering is done by changing the area around the right eye ball. This is an example of malicious distortion or tampering. An effective image hashing scheme should detect this tampering as it has changed the content of the image. To show an example of non-malicious distortion, consider the JPEG compressed version of the Baboon image shown in Figure 2c. In this case, the content of the image is same, however the pixel values would be different. An ideal image hash function should generate similar hash value for images shown in Figure 2a,c as they have similar content. Practical image hash functions however would generate difference hash values. The difference in hash values should be considerably small. A threshold is used to distinguish malicious and non-malicious distortions. Choosing a suitable threshold is still a research challenge in image hashing and is also discussed in this paper.

Devising an image hashing scheme that is robust to non-malicious operations, sensitive to tampering with localization and also secure is quite a challenging task. For example, increasing robustness has a negative effect on tamper detection and security of an image hashing scheme. Image hashing is very much different from cryptographic hash functions, as in the latter, there is no robustness issue. In this paper, a new image hashing scheme is proposed that generates a compact image hash having the ability to detect minute level tampering, offers high robustness to non-malicious distortions and is also secure. To obtain these properties, Gaussian pyramid decomposition of the input image is performed. Although pyramid decomposition has been widely used in image compression [8], computer vision [9], etc., its use in image hashing, as proposed in this paper, has proved to be every effective.

Let us discuss the usefulness of applying Gaussian pyramid decomposition to the input image to obtain a hash which is compact, robust to non-malicious and sensitive to malicious tampering. The input image of size N×M pixels is first resized to 256×256 pixels and then subjected to repeated subsampling and Gaussian filtering to obtain higher levels of the pyramid. Let the resized image of size 256×256 pixels be considered as the base level. The fourth level Gaussian pyramid decomposition of this image would yield a 16×16 matrix. If hash of the input image is constructed using this matrix, it would be compact in size. Due to repeated subsampling and Gaussian filtering, the coefficients at higher levels of the pyramid do not change much when the input image (base level) is subjected to non-malicious distortions. Similarly, if there is malicious tampering in the input image such that the tampered area is greater than a certain percentage of the total image area, there will be change in the corresponding 16×16 matrix coefficient(s). This enables tamper detection with localization. In addition, the hash is made secure using random noise addition to the input image. This makes base level of the pyramid to become a random matrix which is a function of the input image (whose hash is to be calculated) and the random noise which is a function of the secret key shared between the sender and receiver. With this brief overview of the proposed scheme, the main contributions of this paper are as follows:

1. A robust and secure image hashing scheme using Gaussian Pyramids is proposed.
2. A technique of adding random noise is introduced to enforce hash security.
3. The proposed scheme is evaluated using Receiver Operating Characteristics curve (ROC) which demonstrate effectiveness of the proposed scheme under different non-malicious and malicious distortions.

The rest of paper is organized as follows. Section 2 presents review of some image hashing schemes reported in the literature. In Section 3, the effect of secret key on hash security is discussed. Section 4 describes the proposed scheme. Experimental results are presented in Section 5. Finally, Section 6 concludes this paper.

## 2. Literature Review

A number of image hashing schemes have been proposed during the last 15 years to address the core issues discussed in Section 1. Some schemes are focused on tamper detection and reduction of hash size, while others are focused more towards robustness. It is interesting to note that the choice of feature significantly effects the robustness and tamper detection capability of an image hashing scheme. For example, if a feature is robust to geometric distortion and compression, it might be sensitive to contrast enhancement. On the other hand, a feature which is robust to a number of content preserving manipulations might not be sensitive to minute level of malicious tampering. This makes the design of a robust image hashing scheme extremely challenging as there is no defined boundary between content preserving operations and tampering. To get an idea about different features and their impact on image hashing, the following review highlights some image hashing schemes proposed in the literature.

Swaminathan et al. [10] presented a robust and secure image hashing technique using Fourier transform features and controlled randomization. This scheme is robust against a number of non-malicious operations like JPEG compression, RST attacks, shearing, gamma correction, etc. It can also detect cut and paste attacks. Ouyang et al. [11] used Quaternion Discrete Fourier Transforms (QDFT) and Log polar Transform to generate hash for color images. This scheme shows good sensitivity and is robust against content preserving manipulations, especially large angle rotation. Tang et al. [12] proposed an image hashing algorithm using ring partition and non-negative matrix factorization (NMF). This algorithm is robust to image scaling, JPEG compression, contrast adjustment, image rotation, gamma correction, and Gaussian filtering. It shows good discriminative capability against non-malicious manipulations. Tabatabaei et al. [13] introduced a two-stage robust content-based image authentication scheme using Approximate Message Authentication Codes (AMACs). AMACs consists of error correction codes and message authentication codes. Hash generation consists of three steps; pre-processing, intermediate and final hash generation. The results reported in the paper demonstrate high robustness of this scheme against a number of non-malicious operations and is able to detect minute tampering with localization. Ahmed et al. [7] proposed a DWT-based hashing scheme for image authentication which addresses core issues such as tamper detection, robustness and security. The proposed technique yields good robustness against JPEG compression, low-pass filtering and image sharpening. In addition, the scheme can identify small tampering with localization of the tampered regions. However, the scheme is not robust to brightness, contrast enhancement and rotation.

Monga and Mihcak [14] construct an image hash using the idea of non-negative matrix factorization (NMF). They argue that standard rank reduction techniques such as QR factorization and Singular Value Decomposition produce low rank bases which do not necessarily preserve the intrinsic structure of an image. The use of NMF allows computationally simple design with good robustness capability. The hash generated using this method has good discriminative capability when used to compare different images. However it is not clear if small tampered regions can be successfully detected. Zhenjun and Linlin [15] proposed a three-step hashing method to achieve a desirable balance between discrimination and robustness using a technique which they refer to as Local Linear Embedding (LLE). In the first step, the input image is converted into a normalized matrix. The second step consists of constructing a secret key-based secondary image from the normalized matrix obtained from the pre-processed image. Finally, LLE is applied to the secondary image to get low dimensional vectors whose statistics are calculated to produce a short image hash. This scheme provides desirable discrimination and is robust to common signal processing operations.

Xiang et al. [16] constructed a histogram based image hashing scheme. This scheme is robust against geometric deformation. The scheme consists of two steps; firstly, the input image is filtered using a low-pass Gaussian filter. Secondly, a histogram of the preprocessed image is extracted using mean value of the image and a binary string is computed. The hash is secured by making it key-dependent. Experimental results show that the scheme achieves robustness against scaling, rotation, translation and geometric attacks. Similarly, Abbas et al. [17] have also proposed a histogram-based image hashing scheme using Noise Resistant Local Binary Pattern (NRLBP) for achieving robustness and discrimination capabilities. This scheme shows good robustness to a number of content preserving operations and can effectively detect small tampered regions with localization. A disadvantage of this scheme is large size of hash. Tang et al. in [18] use the idea of Color Vector Angle (CVA) to generate image hash. The authors argue in their paper that most of the hashing schemes use luminance information for hash generation of color images. Although computationally less expensive, luminance based features do not always capture details of a color image. To address this issue, Histogram of CVA referred to as HVCA is used as a feature to generate image hash. Results reported in the paper show good discrimination between different sets of images. It appears that this scheme is not designed to detect minute level of tampering with localization.

Zhao and Wei [19] use rotation invariance of magnitudes and corrected phases of zernike moments to generate image hash. This scheme is robust against a number of non-malicious attacks, excluding rotation. It can also detect tampered area with localization. Wu and Niu [20] have proposed an image hashing technique using features extracted by using the Scale Invariant Feature Transform (SIFT). Cyclic matrix are generated from the SIFT features thus translating rotation into elementary transformation of the cyclic matrix. Image hash is obtained by performing Eigen value decomposition of the cyclic matrix. Besides rotation, the proposed scheme is also robust to JPEG compression (QF = 60), scaling, brightness adjustment, minor translation, median filtering and Gaussian noise. The paper however does not show any result exhibiting minute tamper detection and localization capability. In [21], Karsh et al. have proposed an image hashing scheme which is invariant to RST transformations. The hash is constructed using local and global features. Local features are extracted from salient regions of an image using Markov absorption probabilities. Global features are extracted using some statistics measure. The two set of features help to identify small and large tampered regions with good robustness against non-malicious distortions.

Wang et al. [22] proposed a robust hashing scheme for detection of region duplication forgery. This means copying one portion or block of an image and pasting it to another portion or block of the same image. This method is robust to blurring, lossy compression, noise and also copy region rotation. It also reduces total number of blocks and narrows the block matching search space. In [23], Bodin et al. have proposed an image hashing scheme for hybrid document security. This algorithm is capable of securing graphical parts of paper and digital documents with unprecedented performance using a very small image digest. The main contribution of this work is the ability of the hash to work under print and scan noise environment. The input image is first normalized and second-order moments are calculated to form the hash of the image. The image hash contains a number of components such as moments, image resolution, index mapping and color mapping table. Khelif and Jiang [24] proposed a robust and secure hashing algorithm based on virtual watermark detector. The idea proposed by the authors to generate image hash is very interesting. The input image is first pre-processed using a high-pass filter to obtain edge information. The edge-map image is then divided into overlapping blocks and mean of absolute value of coefficients of each block is calculated to form a feature vector. A secret key is used to generate a sequence of virtual watermark. Another secret key is then used to permute the virtual watermark. The permuted virtual watermark and the feature vector coefficients obtained from the input image are given to the watermark detector module. The output of the watermark detector generates hash of the input image. Hash obtained using this scheme is robust to a number of content preserving operations. Results shown in the paper reveals that this scheme cannot detect small level of tampering.

From the above discussion, it is clear that devising an image hashing scheme that is robust to all types of content preserving operations, sensitive to minute level tampering and is also secure is quite a challenging task. Some of the papers reviewed above perform very well for non-malicious distortions but cannot detect minute level tampering. Most of the papers do not secure the hash, which makes it difficult to use hashing for image authentication. If hash is generated without employing a secret key, then an attacker could launch hash collision attacks to defeat the basic purpose of image authentication [7]. On the basis of literature survey, it is concluded that till date, no benchmark has been defined for image hashing schemes because there is always a trade-off between robustness and discriminative capability.

## 3. Effect of Secret Key on Hash Security

Hash is also known as a fingerprint because it is unique for every image. An image hash should also be secure against intentional attacks. In order to achieve this property, Tangent Delay Ellipse Reflecting Cavity Map System (TD-ERCS) [25,26] and Skew tent map [27] have been introduced in the proposed scheme. Mathematically, TD-ERCS is written as [25]:

(1) $$\left\{\begin{array}{cc} x_{n}=-\frac{2k_{n-1}y_{n-1}+x_{n-1}\left(\mu^{2}-k_{n-1}^{2}\right)}{\mu^{2}+k_{n-1}^{2}}\\ y_{n}=k_{n-1}\left(x_{n}-x_{n-1}\right)+y_{n-1}, & n=1,2,3\ldots \end{array}\right.$$

where

(2) $$k_{n}=\frac{2k{}_{n-m}^{\prime }-k_{n-1}+k_{n-1}{\left(k{}_{n-m}^{\prime }\right)}^{2}}{1+2k_{n-1}k{}_{n-m}^{\prime }-k{\left(k{}_{n-m}^{\prime }\right)}^{2}}$$

(3) $$k{}_{n-m}^{\prime }=\left\{\begin{array}{cc} -\frac{x_{n-1}}{y_{n-1}}\mu^{2} & n<m\\ -\frac{x_{n-m}}{y_{n-m}}\mu^{2} & n\geq m \end{array}\right.$$

(4) $$y_{0}=\mu \sqrt{1-x_{0}^{2}}$$

(5) $$k{}_{0}^{\prime }=-\frac{x_{0}}{y_{0}}\mu^{2}$$

(6) $$k_{0}=-\frac{\tan\alpha +k{}_{0}^{\prime }}{1-k{}_{0}^{\prime }\tan\alpha }$$

(7) $$\left\{\begin{array}{c} \mu \in (0,1)\\ x_{0}\in \left[-1,1\right]\\ \alpha \in (0,\pi )\\ m=2,3,4,5\ldots \end{array}\right.$$

Skew tent map is:

(8) $$z_{n+1}=f\left(z_{n},\theta \right)=\left\{\begin{array}{cc} \frac{z_{n}}{\theta }, & \mathrm{if}\;z_{n}\in \left[0,\theta \right]\\ \frac{1-z_{n}}{1-\theta }, & \mathrm{if}\;z_{n}\in \left(\theta ,1\right] \end{array}\right.$$

$x_{n}$, $y_{n}$ and $z_{n}$ are random numbers between 0 and 1. Here $\mu ,x_{0},\alpha$ and *m* are known as seed parameters of TD-ERCS map. In skew tent map, $z_{n}\in \left[0,1\right]$ is the initial value, and θ∈(0,1] is the control parameter. Using $x_{n}$, $y_{n}$ and $z_{n}$, the noisy image is generated using the following pseudo-random number generator (PRNG) equation:

(9) $$\mathrm{PRNG}=\left(x_{n}+x_{y}+z_{n}\right)mod\left(256\right)$$

**Figure 3:**
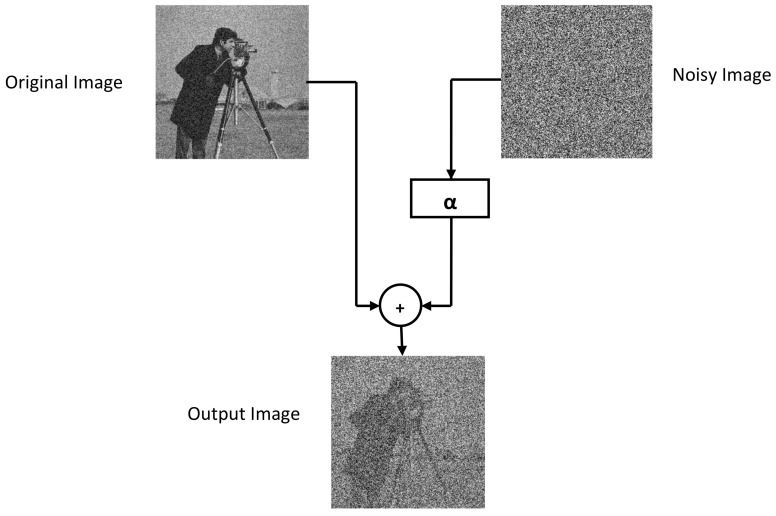
Noisy image added to the original image.

**Figure 4:**
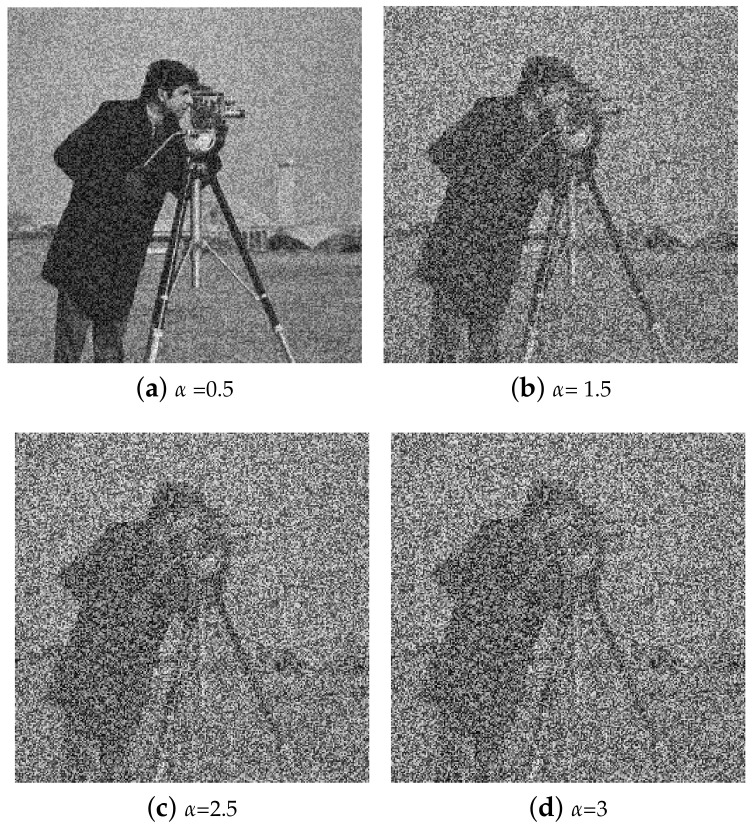
Illustration of Cameraman image at different values of α.

TD-ERCS and Skew tent maps initial parameters are initialized to generate a noisy image having the same dimension as that of the input image. The noisy image is then added to the input image to obtain the output image. Hash of the output image now depends on the input image and the secret key. In other words, the hash feature space is made random. The output image is then processed through Gaussian pyramid decomposition to generate the hash of the input image. Figure 3 shows the perceptual appearance of the original image and the noisy image. As the PRNG is generated using the secret key, therefore the hash depends upon the secret key. By changing the secret key, the hash value will be different. Figure 4 shows the effect of noise on the Cameraman image at different values of α. By increasing the value of α, the effect of noise increases. For example, when α=0.5, the PSNR was 17.87 dB, when α=1.5, the PSNR was 12.76 dB. Similarly for α=2.5 and α=3, the observed PSNR was 11.25 dB and 10.83 dB, respectively. Therefore, by increasing the value of α, the generated hash will become more random thus making it difficult for an attacker to guess the hash value and launch hash collision attacks. In addition, for the same image, different hash values can be generated by changing the secret key.

## 4. The Proposed Scheme

**Figure 5:**
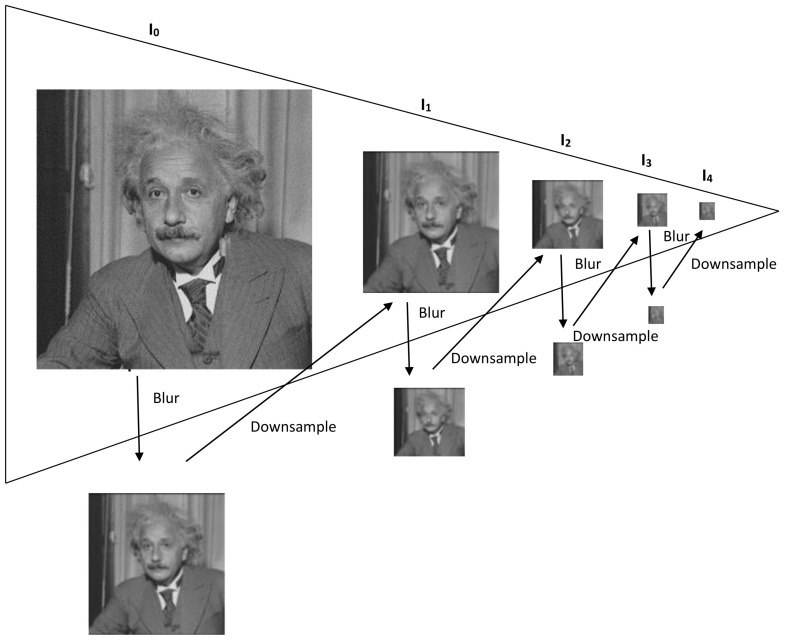
Illustration of Gaussian Pyramid.

A new image hashing scheme is proposed in this paper which simultaneously attempts to address the core issues of robustness to non-malicious distortions, detection of minute level tampering and securing the hash using a secret key. This is accomplished by obtaining Gaussian pyramid decomposition of the input image. An illustration of a 4-level Gaussian pyramid is shown in Figure 5. The input image is considered as the base level, $I_{0}$. The upper level of the pyramid is obtained by down-sampling and low-pass filtering the previous level. Let $I_{1}$, $I_{2}$, $I_{3}$ and $I_{4}$ represent the first, second, third and fourth levels of the Gaussian pyramid, respectively. As an example, suppose the image $I_{0}$ has a dimension of 256×256 pixels. The entries in $I_{4}$ will therefore be 16×16. There will be only 256 entries in $I_{4}$ as compared to 65,536 entries in $I_{0}$. Therefore, the hash generated by using $I_{4}$ will be compact in size. In addition, the entries in $I_{4}$ do not change significantly due to non-malicious manipulations like filtering, compression, etc. However, there is a significant change in an $I_{4}$ entry whose corresponding spatial area in $I_{0}$ has been maliciously tampered. This explains the reason behind the proposed scheme to be robust against non-malicious distortions and sensitive to detect tampering with localization. To further reduce hash size, disk and LOG filters are applied to the top level of the pyramid to obtain two different matrices. The difference of these two matrices is the hash of the input image $I_{0}$. Since both these filters perform blurring operation to $I_{4}$, therefore the entries in the difference matrix are small in magnitude as compared to entries in $I_{0}$. In fact, some of entries in $I_{4}$ are very close to zero, thus significantly reducing hash size. The value of σ for the LOG filter is 0.5 and the value of radius for the disk filter is 2. These parameters have been obtained after thorough experimentation involving a number of non-malicious distortions and different types of tampering cases. The proposed scheme consists of hash generation and hash verification modules which are now explained.

### 4.1. Hash Generation Module

**Figure 6:**
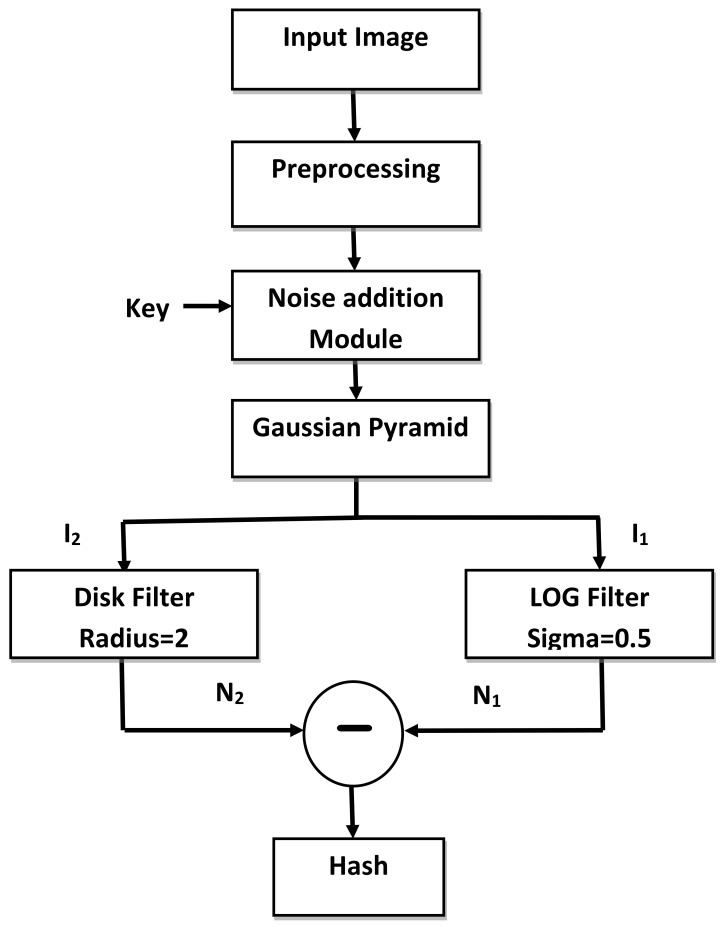
Block diagram of hash generation module.

The block diagram of hash generation module is given in Figure 6 and the steps are mentioned below.

1. The input image I(x,y) of dimension M×N is converted to a gray scale image and then resized to 256 × 256 pixels. The notation *x*,*y* is used to represent the spatial location of a pixel. Let $I_{0}\left(x,y\right)$ represent the normalized image which is given by Equation (10).
  (10) $$I\to \;I_{o}$$
2. Let *k* be the secret key which is shared between the sender and the receiver through a secure channel. To enforce security in the hash, a noisy image is added to $I_{0}$ to obtain $I_{N}$.
  (11) $$I_{N}\left(x,y\right)=I_{0}\left(x,y\right)+\left(\alpha *N_{k}\right),$$
  where α controls the intensity of noise. The notation $N_{k}$ refers to the noisy image which is generated using the secret key *k*. A pseudo-random number is initialized using *k* to generate the noisy image.
3. The image $I_{N}$ is decomposed to level 4 using Gaussian pyramid decomposition to get a 16 × 16 matrix represented by $I_{4}$. Level 3 decomposition would generate hash of a larger size, whereas level 5 decomposition would yield hash features that would not be sensitive enough to detect minute level tampering. After experimentation, level 4 Gaussian pyramid decomposition for a 256 × 256 image was found to be the best choice.
4. The image $I_{1}$ is then filtered using LOG and disk filters. As for the LOG filter, the value of α is 0.5 and the window size is 5×5. Let $N_{1}$ be the output image of the LOG filter. The size of radius for the disk filter is equal to 2. If value of the radius increases, blurring will increase. The output image of disk filter is represented by $N_{2}$.
5. $N_{1}$ and $N_{2}$ are subtracted and their difference is the final hash H of the input image *I*.

(12) $$H=N_{1}-N_{2}$$

### 4.2. Hash Verification Module

**Figure 7:**
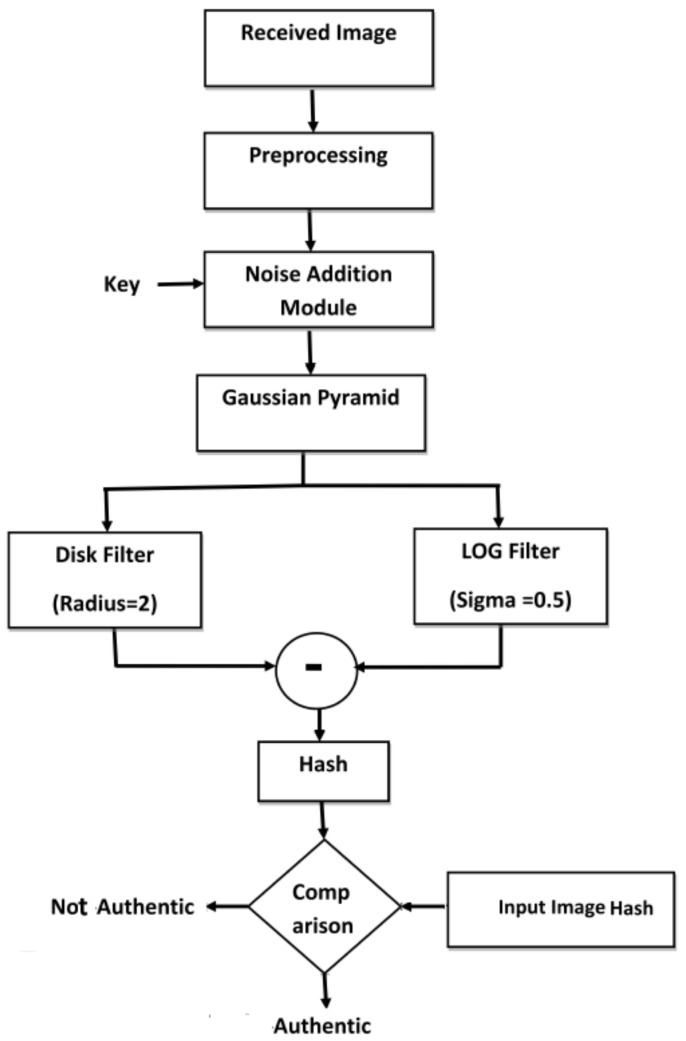
Block diagram of hash verification module.

The hash verification module shown in Figure 7 is used to verify the authenticity of the received image, $I^{\prime }$.

1. The received image $I^{\prime }$ of dimension M×N is processed through the same steps as discussed in the previous section to obtain hash of the received image, $H^{\prime }$.
2. A difference matrix *d* is calculated as shown by Equation (13)
  (13) $$d=H-H^{\prime }$$
3. Each element of *d* is compared with a chosen threshold $T_{h}$. If any element of *d* is greater than $T_{h}$, then the corresponding spatial area of the image would be considered as tampered.

## 5. Experimental Evaluation

As discussed in Section 1, the quality of a good PHF scheme is that it should detect minute level tampering and at the same time be robust to non-malicious distortions. Perceptual robustness accounts for content preserving manipulations such as Gaussian noise, blurring, JPEG compression, scaling, etc. The difference between two hashes should be ideally zero or very small for two similar images even if one of the image undergoes non-malicious distortion. Similarly, the difference between hash of one image and its tampered version should be large to detect tampered regions. A number of results are presented in this section to demonstrate both these cases. In addition, the Receiver Operating Characteristics Curve (ROC) analysis is also presented. The ROC curve is a plot between False Positive Probability (FPP) and False Negative Probability (FNP). False Positive Probability shows the number of tampered images verified as genuine. Similarly the probability to discard an original image and detect it as a tampered image is called False Negative Probability. To evaluate robustness and tamper detection capability of the proposed scheme, several test images are used. Let $d_{max}$ denote the maximum value of the *d* matrix. In case of non-malicious distortion, the value of $d_{max}$ should be less than the chosen threshold.

### 5.1. Tamper Detection Test

**Figure 8:**
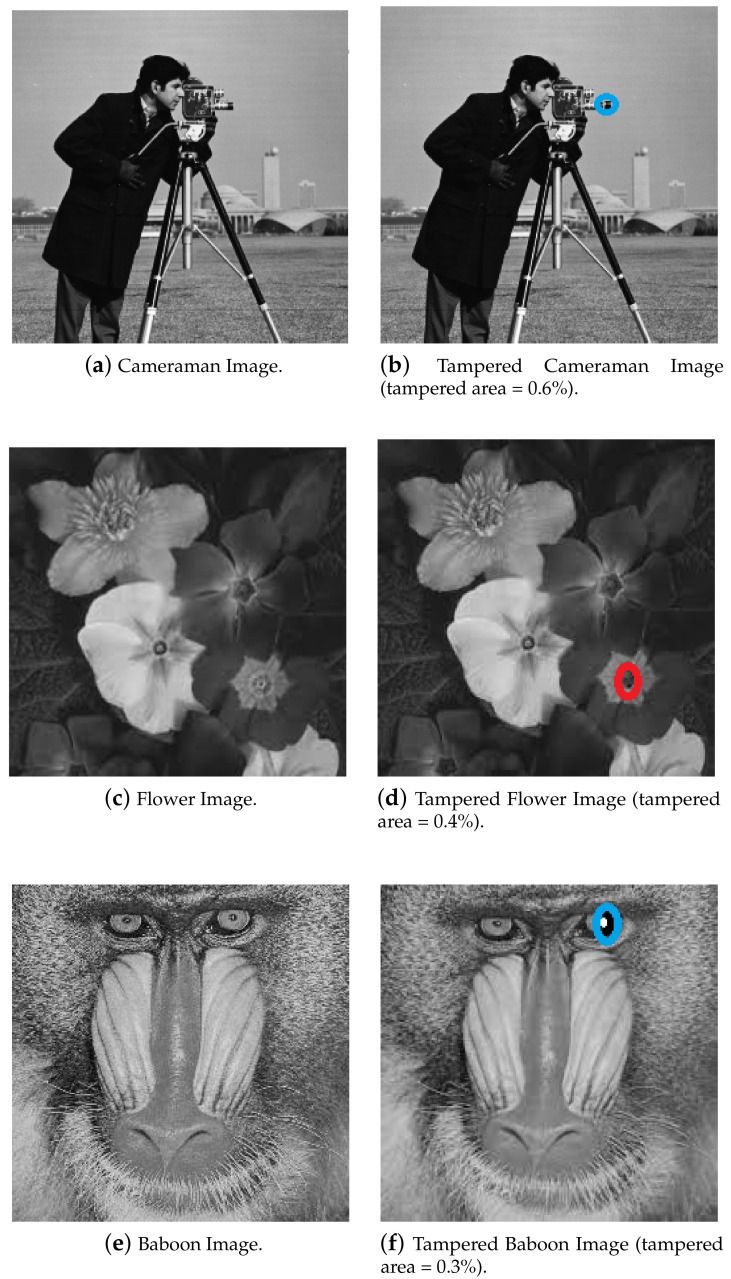
Test images and their tampered versions. (Tampering is shown inside the circle.)

To check the tamper detection capability of the proposed scheme, the Einstein image, Cameraman image, Flower image, Joker image, Girl and Baboon images were considered as test images. Three test images and their tampered versions are shown in Figure 8. It is important to mention that for all the tampered images under consideration, the tampered area is less than 1% of the total area of the respective original image. The tampered area considered in this paper is small when compared with other papers, for example, [28]. Hashes of original images and their respective tampered versions were calculated and the difference matrix is calculated to obtain $d_{max}$. The result of $d_{max}$ is tabulated in Table 1. This result reveals that if the value of threshold $T_{h}$ is kept at 20, then all the tampered cases would be successfully detected.

### 5.2. Robustness and Threshold Selection

Robustness of an image hashing scheme plays a vital role in case of content preserving operations such as Gaussian noise, speckle noise, motion blurring, average blurring, JPEG compression, etc. It is required to obtain distant hashes for perceptually different images. To check robustness of the proposed scheme, hash of the original image was compared with the hash of the distorted versions of the same image. The type of manipulations and their parameters are listed in Table 2. These parameters were obtained after doing experiments on different images and observing the degradation level. The degradation level was kept such that the structure of the image remains intact. The value of $d_{max}$ for non-malicious distortions is shown in Table 3. From this result, it is evident that the maximum value of $d_{max}$ for non-malicious distortions is 13.5 whereas the minimum value of $d_{max}$ for tampering as shown in Table 1 is 20. There is a clear boundary separating non-malicious manipulation and tamper detection for the test images under consideration. These results reveal that the proposed scheme is robust to non-malicious distortions and sensitive to minute tampering. A suitable threshold needs to be identified in order to differentiate malicious and non-malicious distortions. Since the maximum value of $d_{max}$ is 13.5 for non-malicious distortions and the minimum value of $d_{max}$ is 20 for tampering, therefore a threshold between 14 and 19 can be used to distinguish tampering from non-malicious distortions.

### 5.3. Receiver Operating Characteristics (ROC) Curves

Performance of the proposed scheme is evaluated using the Receiver Operating Characteristics (ROC) Curve. The ROC curve is a plot between False Positive Probability (FPP) and False Negative Probability (FNP) w.r.t to changing threshold. These two probabilities are given by Equations (14) and (15), respectively. A higher value of FPP means tampered images will be verified as genuine. Similarly a system with higher FNN would reject genuine images. There is a need to balance robustness and tamper detection capability by selecting a suitable threshold. In an image hashing system, the operating point of the ROC curve depends upon the desired application. The ROC operating point should be chosen such that FPP and FNP are small, thus making the system reliable.

(14) $$FPP=(N_{T}^{A})/NT$$

(15) $$FNP=(N_{A}^{T})/NA$$

$N_{T}^{A}$ in Equation (14) shows the total number of tampered image blocks detected as authentic, whereas NT represents the total number of tampered image blocks. Similarly in Equation (15), $N_{A}^{T}$ shows the total number of authentic image blocks detected as tampered and NA represents the total number of authentic image blocks.

**Figure 9:**
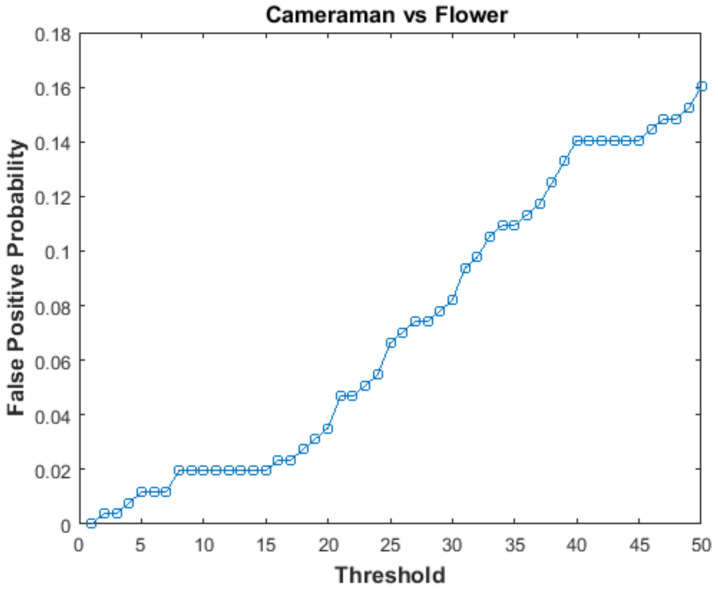
False Positive Probability vs threshold.

In order to calculate FPP, hash of the Flower image was compared with hash of the Cameraman image. These two images were chosen as they are perceptually different. By changing the value of threshold $T_{h}$, positively authenticated image blocks were observed and FPP was calculated using Equation (14). The graph of FPP versus threshold is shown in Figure 9. The value of FPP increases with the increase in threshold ($T_{h}$). At $T_{h}$ = 20, the value of FPP is 0.03, which is good.

To estimate FNP, non-malicious distortions, i.e., Gaussian noise, speckle noise, Gaussian blurring, motion blurring, average blurring, image sharpening and JPEG compression were applied to obtain distorted images. Hashes of distorted images were compared with hashes of their respective original images. The distortion parameters are shown in Table 2. To plot FNP, the threshold value was varied from 0 to 50. For all the cases, the value of FNP becomes zero when the value of threshold is greater than 15. This is a very encouraging result as it is consistent with the threshold selection methodology discussed in Section 5.2. From these results, it is evident that the proposed image hashing scheme is highly robust to a number of non-malicious distortions and capable of detecting minute level of tampering.

**Figure 10:**
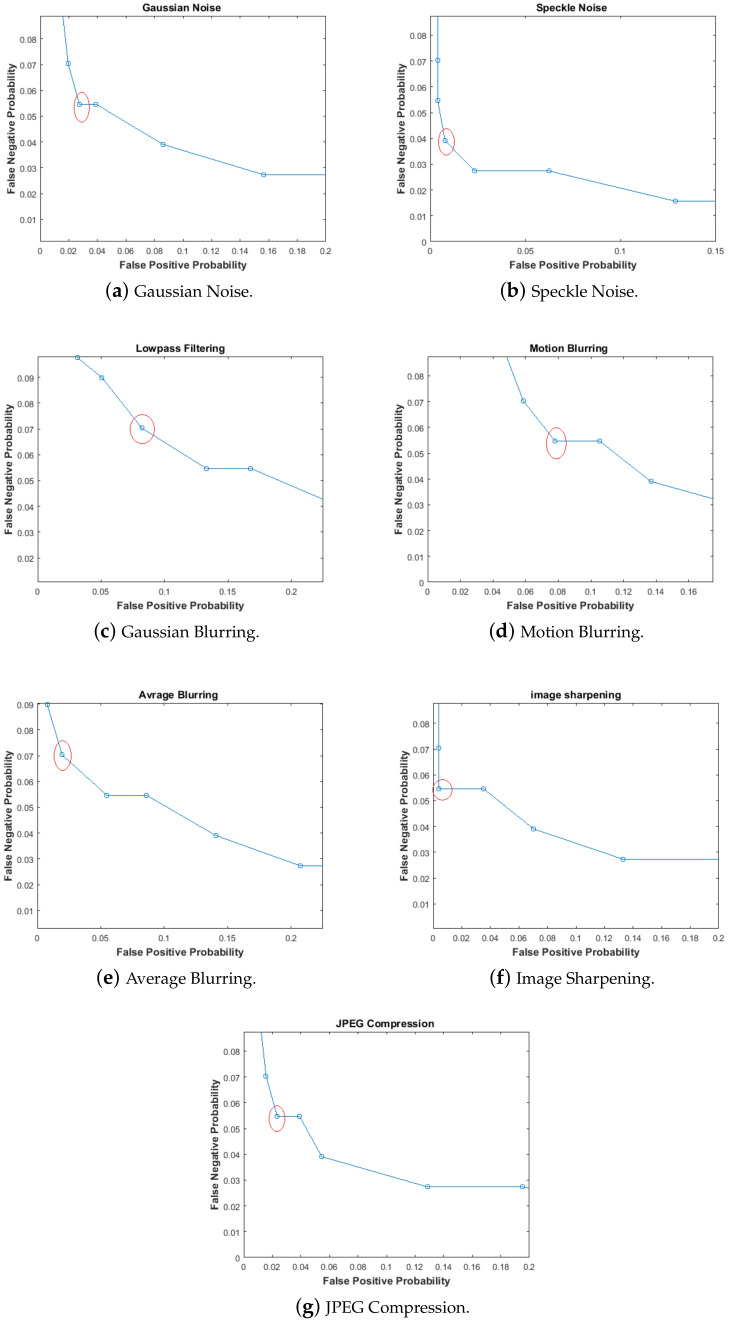
Receiver Operating Characteristics (ROC) Curves.

After FPP and FNP estimation, finding the ROC curves becomes easy. The ROC curve for the proposed system is plotted by taking FPP on the x-axis and FNP on the y-axis. It is required that either both FPP and FNP be small enough to be negligible or at least FPP should be very small if not possibly zero. Both the probabilities have an inverse relationship. The ROC curve gives an idea about the selection of a suitable threshold by selecting minimum values of FPP and FNP. The ROC curves for different non-malicious distortions are shown in Figure 10. The values of FPP and FNP are tabulated in Table 4. These results reveal that the proposed scheme has low FNN at low FNP.

### 5.4. Comparison of ROC Curves with Other Schemes

Analysis of ROC curves of the proposed scheme show promising results for tamper detection and non-malicious distortions. In Table 5, FPP and FNP of the proposed scheme is compared with some state-of-the-art schemes [10,17,23,24,28,29] in image hashing. The first four papers are among the most cited in the literature and the last two papers are recently published. Although tampering in these papers is greater than the tampering used in the tests of proposed scheme, yet the proposed scheme shows very good results. FPP and FNP of the under consideration papers and of the proposed scheme are mentioned in Table 5. In these papers, the result of non-malicious distortions were not shown separately rather the cumulative effect of all distortions was used to find FNP and FPP.

From the results mentioned in Table 5, it is observed that for Gaussian blurring, average blurring and JPEG compression, the proposed hashing technique gives better results in terms of FPP and FNP values.

### 5.5. Effect of Noise on Hash Size

The size of hash depends upon the concentration of noise, α. To check the effect of α on the size of hash, the value of α was varied from 0 to 3. Table 6 shows the effect of noise and the corresponding maximum value of hash coefficients for three test images. When the value of α is between 0 and 1, the maximum value of hash coefficient for all the test images is 433. This means that each element hash would require 9 bits of storage. Since the hash size of the proposed scheme is 16 × 16, the total hash would require 2304 bits or 288 bytes. Similarly when α was varied from 1.5 to 3, the maximum value of hash coefficient for all the test images was found to be 712 which require 10 bits of storage. Hence the size of hash for an image of size 256 × 256 would require 320 bytes. Since there is not much difference between the maximum values of hash when noise is increased from 0 to 3, therefore, the proposed algorithm can generate secure hash without significant increase in the size of hash.

### 5.6. Effect of Noise on Malicious and Non-Malicious Distortions

It is important to check whether the addition of noise to enforce security in any way changes the value of $d_{max}$ for malicious and non-malicious distortions. The flower image and its tampered version were taken as a test case. For non-malicious distortion, JPEG compression was considered. Table 7 shows the results for different values of α. It is evident from this result that noise addition does not change robustness and tamper detection capability of the system.

### 5.7. Effect of Noise on System’s Security

It is a necessary requirement of security that when the key is changed, there should be a significant change in the hash value. To find the effect of noise on the security of the system, two different keys were applied on the same image and the value of $d_{max}$ was calculated which is tabulated in Table 8. As the value of α increases, the value of $d_{max}$ increases thus demonstrating that noise concentration has a profound effect on the security of the system. For example, if noise value is taken as 2, the value of $d_{max}$ is 95, which is far greater than the threshold range. Therefore, if two different keys are applied on the same image, the corresponding hash values will be different. To generate the required hash, the correct key needs to be used.

### 5.8. Key Sensitivity and Key Space Analysis

**Figure 11:**
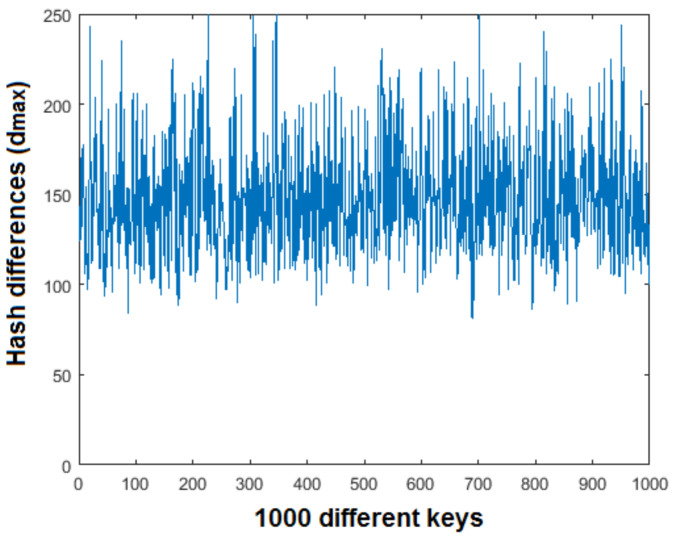
Effect of secret key on the Hash.

To check sensitivity of the secret key, an experiment was performed in which 1000 different keys were used on the Baboon image. The result of this experiment is shown in Figure 11 depicting the maximum and minimum change in the value of $d_{max}$, which is 264 to 81, respectively. This demonstrates that the proposed hashing scheme is very sensitive to change in the secret key. The key space of an algorithm is the set of all possible keys. In a good cryptosystem, the key space must be large enough to resist cryptographic attacks. For the TD-ERCS and Skew tent maps outlined in Section 3, one can see that the key size is dependent on 6 different initial conditions. For a computational precision of around $10^{-15}$, the key space is $10^{90}$ which is approximately equal to $2^{299}$. This key space is high enough to resist brute force attacks and is comparable to state-of-the-art text hashing schemes, for example, [30,31].

## 6. Conclusions

In this paper, an image hashing scheme using Gaussian pyramid is proposed and evaluated. Using Gaussian pyramid technique, good robustness characteristics have been achieved along with minute level of tamper detection. The proposed scheme uses fourth level Gaussian pyramid decomposition to generate a hash size of 16 × 16, thus obtaining a compact hash consisting of only 256 entries for an image of size 256×256. To enhance security of the algorithm, random noise is added to the input image thus making the entire hash feature space random. The generated hash is therefore a function of the input image and the random noise. This makes it difficult for an attacker to predict the hash for a given input image. The proposed image hashing scheme was evaluated using a number of experiments involving malicious and non-malicious distortions. The results of these experiments helped to select a suitable threshold thus defining a clear boundary between non-malicious distortions and tampering. It is encouraging to note that the proposed hashing scheme has low FPP and FNP values when compared with state-of-the-art image hashing algorithms proposed in the literature. Since the hash is key dependent, the proposed scheme can be used in image authentication, which, besides robustness and tamper detection, also demands security of a hash function.

## Figures and Tables

**Table 1:** Value of $d_{max}$ when hashes of the original image and its respective tampered version are subtracted.

Tampered Images	Maximum Difference
Flower image	43
Joker image	122
Baboon image	31
Einstein image	36
Girl image	135
Baboon image	32
Cameraman image	20

**Table 2:** Non-malicious distortion parameters.

Distortion Type	Details	Control Parameters	Specifications
Noise	Gaussian Noise	Mean (*m*), Variance (*v*)	*m* = 0, *v* = 0.007
	Speckle Noise	Noise Variance (*Nv*)	*Nv* = 0.01
Blurring	Gaussian Blurring	Standard Deviation (σ),	σ = 3.5,
		Window size (*Fs*)	*Fs* = [9×9]
	Motion Blurring	Linear Motion by pixel (*L*),	*L* = 10,
		Angle (θ)	θ = 90
	Average Blurring	Radius r	r = 4
Luminance Changes	Gamma Correction	Gamma (γ)	γ = 1.2
	Image Sharpening	Radius (*r*),	*r* = 2,
		Sharpening amount (Fh)	Fh=1
Geometric Attacks	JPEG Compression	Quality factor (*Q*)	*Q* = 10

**Table 3:** Value of $d_{max}$ for Flower, Cameraman and Baboon images.

Non Malicious Distortions	Difference		
	**Flower Image**	**Cameraman Image**	**Baboon Image**
Gaussian Noise *m* = 0, *v* = 0.007	9.3	10.54	12.8
Speckle Noise Nv = 0.01	5.5	7.35	8.3
Gaussian Blurring σ = 3.5	4.09	6.99	7.8
Motion Blurring *L* = 10, θ = 90	9.44	6.87	8.27
Average Blurring *r* = 4	8.5	9.7	6.08
Image Sharpening *r* = 2, Fh = 1	9.89	13.5	5.11
JPEG Compression *Q* = 10	10.2	11.5	8.7

**Table 4:** Values of False Positive Probability (FPP) and False Negative Probability (FNP) for non-malicious distortions.

Non Malicious Distortions	FPP	FNP
Gaussian Noise *m* = 0, *v* = 0.007	0.02	0.055
Speckle Noise Nv = 0.01	0.01	0.04
Gaussian Blurring σ = 3.5, Fs = [9×9]	0.07	0.07
Motion Blurring *L* = 10, θ = 90	0.07	0.055
Average Blurring *r* = 4	0.02	0.07
Image Sharpening *r* = 2, Fh = 1	0.01	0.055
JPEG Compression *Q* = 10	0.02	0.055

**Table 5:** Values of FPP and FNP for non-malicious distortions.

Other Schemes	FPP	FNP
Monga [28]	0.02	0.03
Swaminathan [10]	0.05	0.05
Khelif [24]	0	0.09
Kim [29]	0.09	0.07
Abbas [17]	0.01	0.01
Eskenazi [23]	0.09	0.01
**Proposed Scheme**	**FPP**	**FNP**
Gaussian Noise *m* = 0, *v* = 0.007	0.02	0.055
Speckle Noise Nv = 0.01	0.01	0.04
Gaussian Blurring σ = 3.5, Fs = [9×9]	0.07	0.07
Motion Blurring *L* = 10, θ = 90	0.07	0.055
Average Blurring *r* = 4	0.02	0.07
Image Sharpening *r* = 2, Fh = 1	0.01	0.055
JPEG Compression *Q* = 10	0.02	0.055

**Table 6:** Effect of noise on hash size.

Noise Value	Maximum Value of Hash Coefficient		
	**Cameraman Image**	**Flower Image**	**Baboon Image**
0	294	310	262
0.5	360	371	328
1	426	433	396
1.5	492	503	464
2	558	572	532
2.5	624	642	602
3	691	712	672

**Table 7:** Values of $d_{max}$ after applying JPEG compression and tampering to flower image.

Noise Value	Value of $d_{\max}$ after applying JPEG Compression	Value of $d_{\max}$ after Tampering
0	11	43
0.5	10	43
1	10	43
1.5	10	43
2	10	43
2.5	10	43
3	10	43

**Table 8:** Effect of noise on system security for different keys.

Noise Value	Maximum Value of Hash Coefficient $d_{\max}$
0	0
0.5	24
1	48
1.5	72
2	95
2.5	119
3	143
